# Brain microglia were activated in sporadic CJD but almost unchanged in fatal familial insomnia and G114V genetic CJD

**DOI:** 10.1186/1743-422X-10-216

**Published:** 2013-07-01

**Authors:** Qi Shi, Wu-Ling Xie, BaoYun Zhang, Li-Na Chen, Yin Xu, Ke Wang, Ke Ren, Xiao-Mei Zhang, Cao Chen, Jin Zhang, Xiao-Ping Dong

**Affiliations:** 1 State Key Laboratory for Infectious Disease Prevention and Control, Collaborative Innovation Center for Diagnosis and Treatment of Infectious Diseases (Hangzhou), National Institute for Viral Disease Control and Prevention; Chinese Center for Disease Control and Prevention, Chang-Bai Rd 155, Beijing 102206, People’s Republic of China; 2 Chinese Academy of Sciences Key Laboratory of Pathogenic Microbiology and Immunology, Institute of Microbiology, Chinese Academy of Sciences, Beijing 100101, China

**Keywords:** Prions, Microglia, Creutzfeldt-Jakob disease, Fatal familial insomnia, G114V, Cytokines

## Abstract

**Background:**

Microglial activations have been described in different subtypes of human prion diseases such as sporadic Creutzfeldt-Jakob disease (CJD), variant CJD, Kuru and Gerstmann-Sträussler-Scheinker disease (GSS). However, the situation of microglia in other genetic prion diseases such as fatal familial insomnia (FFI) and familial CJD remains less understood. The brain microglia was evaluated comparatively between the FFI, G114V and sCJD cases in the study.

**Methods:**

Specific Western blots, immunohistochemical and immunofluorescent assays were used to detect the changes of microglia and ELISA tests were used for levels of inflammatory cytokines.

**Results:**

Western blots, immunohistochemical and immunofluorescent assays illustrated almost unchanged microglia in the temporal lobes of FFI and G114V gCJD, but obviously increased in those of sCJD. The Iba1-levels maintained comparable in six different brain regions of FFI and G114V cases, including thalamus, cingulate gyrus, frontal cortex, parietal cortex, occipital cortex and temporal cortex. ELISA tests for inflammatory cytokines revealed significantly up-regulated IL-1β, IL-6 and TNF-α in the brain homogenates from sCJD, but not in those from FFI and G114V gCJD.

**Conclusion:**

Data here demonstrates silent brain microglia in FFI and G114V gCJD but obviously increased in sCJD, which reflects various pathogenesis of different human prion diseases subtypes.

## Introduction

Human prion diseases or human transmissible encephalopathies (TSEs) are fatal neurodegenerative disorders including Kuru, Creutzfeldt-Jakob disease (CJD), Gerstmann-Sträussler-Scheinker disease (GSS) and fatal familial insomnia (FFI). They can be sporadic, e.g. sporadic CJD (sCJD), inherited, e.g. genetic CJD (gCJD), GSS and FFI, or acquired by infection, e.g. Kuru, iatrogenic CJD (iCJD) and variant CJD (vCJD). Although human TSEs are all related with a special agent, named prion, the clinical manifestations, neuropathological abnormalities and molecular traits of prions may vary largely among various human prion diseases [1].

Microglial cells are resident mononuclear phagocytes in central nervous system (CNS) and comprise approximately 10% of the adult CNS total cell population. Normally microglia functions as immunocompetent and phagocytic cells, which distribute throughout CNS as a network of resting but readily responsive cells. In disease situations, microglial cells possess an extremely plastic chameleon-like phenotype, which respond sensitively to pathological challenges [2]. Upon activation, they transform into phagocytes and release a range of substances, such as cytokines/chemokines, nitric oxide, free radicals and neurotrophic molecules. Earlier studies have illustrated activation of microglia in the brains of human prion diseases, including sCJD, Kuru and GSS, where more frequently observed in GSS, followed by Kuru and less predominant in sCJD [3,4]. Analysis of mRNA expression in three rodent models of CJD confirmed remarkable differences in the pattern of glial activation in these models [5]. A comprehensive study of 26 sCJD cases even proposes that strong glial activation is associated with type 1 PrP^Sc^ and diffuse PrP immunoreactivity, while mild glial reaction with type 2 PrP^Sc^ and focal PrP deposits [6]. However, the situation of microglia in other human genetic prion diseases, such as FFI and fCJD, remains less understood.

In this report, we comparatively analyzed the brain microglia in temporal lobes of three FFI cases, a G114V gCJD case and two sCJD cases. Obvious activation of microglia was observed in two sCJD brains, either with type 1 and type 2 PrP^Sc^, but almost not in FFI and G114V cases. Meanwhile, the activation of microglia is assumed not to be specifically related spongiform degeneration, deposits of PrP^Sc^, neuron loss and astrogliosis. Our data here indicate that the microglia reaction vary largely depending on the different human prion agents.

## Results

**Figure 1:**
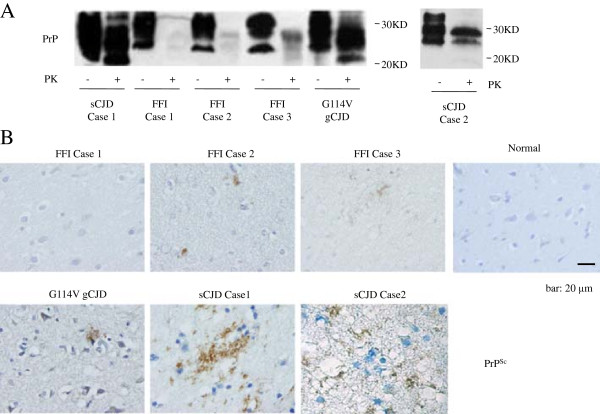
**PrP**^**Sc **^**deposits in the tissues of temporal lobes of different human prion diseases. A**. Western blots. Same amounts (10 μl) of 10% brain homogenates from three FFI, two sCJD and one G114V gCJD were loaded into 15% SDS-PAGE, after treated with 20 μg/ml PK (+) or without PK (−). Molecular markers are indicated on the right. **B**. Immunohistochemical assays. Tissue sections from three FFI, one sCJD (Case 1), one G114V gCJD and one normal control were strained with PrP mAb 3F4 after treated with 6M GdnHCl. Different prion diseases and normal control are indicated above. Scale bar, 20 μm.

The temporal lobes of seven human prion diseases were enrolled into the assays for PrP^Sc^, among them, one was biopsy specimen (sCJD Case 2) and the rest were postmortem ones. PK-digested Western blots showed clear three PrP-specific bands in the tissue homogenates of G114V gCJD and two sCJDs, while only very weak signals in that of three FFI cases (Figure 1A). As expected, the monoglycosyl PrP^Sc^ band in G114V gCJD and diglycosal PrP^Sc^ bands in FFI were predominant, respectively. The profiles of PrP^Sc^ signals of two sCJD cases seemed to be different, with predominate monoglycosyl PrP^Sc^ band and relatively lager aglycosal band (>20 KD) in sCJD Case 1, whereas with predominate diglycosyl PrP^Sc^ band and relatively small aglycosal band (<20 KD) in sCJD Case 2 (Figure 1A). To see the deposits of PrP^Sc^ in brain tissues, the sections of temporal lobes of those patients were stained with PrP-specific mAb 3F4 after treatment of GdnHCl. As shown in Figure 1B, lager amounts of PrP^Sc^ accompanying with different sizes of vacuolation were detected in the sections from two sCJD cases and G114V gCJD, while only a few PrP^Sc^ signals were observed in those of FFI Case 2 and 3, and almost no PrP^Sc^ in FFI Case 1.

### Increased Iba1 levels in the brain tissues of sCJD cases, but not in that of the cases of FFI and G114V gCJD

**Figure 2:**
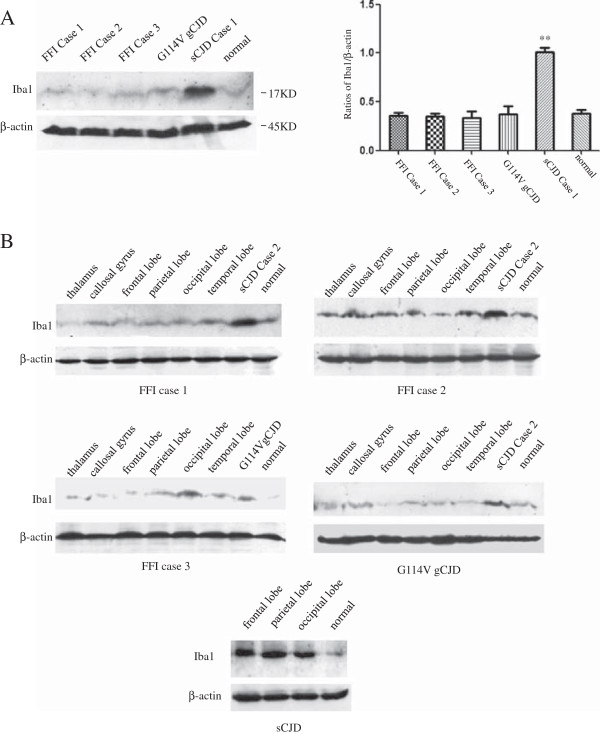
**Evaluation of the brain Iba1 levels of different human prion diseases by Western blots. A**. Temporal lobes. Same amounts of 10% homogenates from three FFI, one sCJD (Case 1), one G114V gCJD and normal control were separated onto 15% SDS-PAGE and blotted with Iba1 or β-actin specific mAbs. The quantitative analyses of the relative gray values are showed on the right. The results are calculated from three blots and presented as mean ± SD. Statistical differences are illustrated as **P<0.01. **B**. Different brain regions. Same amounts of 10% homogenates of six regions, including thalamus, cingulate gyrus, frontal lobe, parietal lobe, occipital lobe and temporal lobe, from three FFI and the G114V gCJD cases were comparatively assayed. Same amounts of 10% homogenates of temporal lobe from one sCJD (Case 2) and normal one were loaded as control. Same amounts of 10% homogenates of frontal lobe, parietal lobe and occipital lobe from sCJD (Case 1) were also comparatively assayed with that of normal control (low panel). Molecular markers are indicated on the right.

To see the potential change in microglia, the levels of Iba1 that was commonly used as the marker of total microglia including resting and activated state in temporal lobes were comparatively evaluated by Western blots. Compared with that of normal control, the Iba1 signal in sCJD (Case 1) was remarkably stronger (P<0.01), while that in the FFI and G114V gCJD maintained almost unchanged (Figure 2A). To test the Iba1 levels in other brain regions, six different regions including thalamus, callosal gyrus, frontal lobe, parietal lobe, occipital lobe and temporal lobe from three FFI cases and a G114V gCJD case were subjected into Iba1-specific Western blots, together with temporal lobe from sCJD Case 2 and normal control. It showed that the Iba1 signals in the tested six regions from FFI and G114V gCJD cases were fairly comparable, revealing similar signal intensities as that of normal control, whereas the intensities of Iba1 signals in sCJD Case 2 were significantly stronger (Figure 2B). Moreover, the Iba1 signals in three brain regions of sCJD (Case 1) were significantly stronger than that of normal control (Figure 2B). It indicates that the level of brain Iba1 increased in sCJD cases, but not in FFI and G114V gCJD cases.

### Larger microglial cells in the brains of sCJD cases, but not in that of the cases of FFI and G114V gCJD

**Figure 3:**
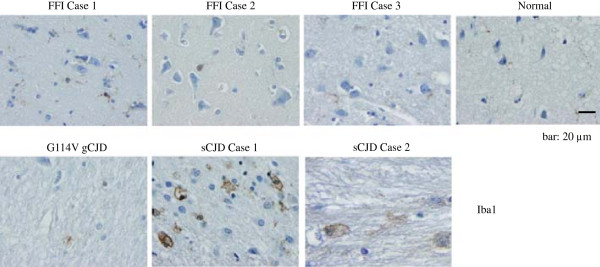
**Immunohistochemical assays of microglia in the temporal lobes of three FFI, two sCJD, the G114V gCJD and normal control with Iba-1 specific antibody.** Different prion diseases and normal control are indicated above. Scale bar, 20 μm.

To reveal the morphological difference in microglia among various subtypes of human prion diseases, the sections of temporal lobes from three FFI cases, two sCJD cases, one G114V gCJD and a normal control were employed into Iba1 specific IHC assays. Abundances of microglia with much larger round- or amoeboid-shape cell bodies were observed in the brain sections of two sCJDs, but only a few Iba1 positive-stained cells with small cell body were detected in the tested two kinds of inherited prion diseases showing similar patterns as normal one (Figure 3), even in the brains of G114V gCJD which contained lager amounts PrP^Sc^ deposits. This kind of enlarged morphology of microglia is usually thought to be associated with an activated and phagocytic state [2], which may suggest an enhanced microglia population in the CNS tissues of sCJD.

**Figure 4:**
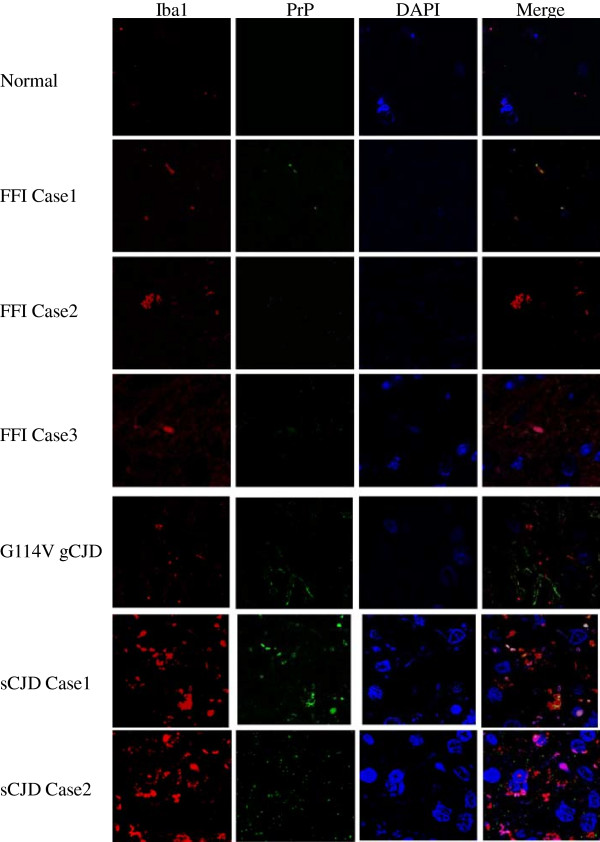
**Immunofluorescent assays of microglia (with Iba-1 specific antibody) and PrP**^**Sc **^**(with PrP specific antibody) in the temporal lobes of different human prion diseases.** The images of PrP^Sc^ (green), Iba-1 (red), DAPI (blue) and merge are monitored under a confocal microscopy and indicated above. Various prion diseases and normal control are indicated on the left.

Furthermore, Iba1- and PrP-specific double-stained immunofluorescent assays were conducted with the brain sections of various human prion diseases after treatment with GdnHCl. Confocal microscopy illustrated more PrP^Sc^ specific signals (green) in the brain tissues of sCJD cases, less amounts in G114V gCJD, whereas almost undetectable in FFI cases (Figure 4) Meanwhile, obviously large amounts of Iba1 specific signals (red) were observed in the brain sections of sCJD cases, but clearly less in that of FFI cases and G114V gCJD (Figure 4), which highlights an active proliferation of microglia specially in the brain tissues of sCJD.

### Activation of brain microglia seemed not to be related with the special neuropathological changes of prion diseases

**Figure 5:**
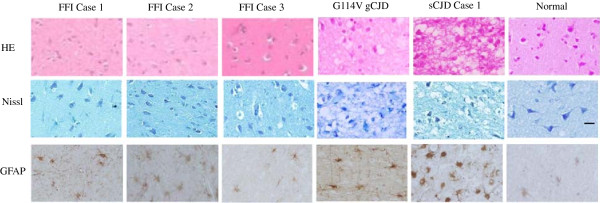
**Neuropathological assays of temporal lobes of three FFI cases, a sCJD (Case 1) patient, a G114V gCJD patient and normal control (indicated above).** Upper: HE stain. Middle: Nissl stain. Bottom: GAFP-specific IHC. Scale bar, 20 μm.

To see the linkage of the activation of microglia with prion associated neuropathological changes among various subtypes of human prion diseases, the sections of temporal lobes from three FFI cases, the G114V gCJD and a sCJD case (Case 1) were comparatively analyzed. HE stainings revealed large amounts of spongiform degenerations in the brains of sCJD and G114V gCJD (Figure 5, upper panel). Nissl stainings showed obvious pyknotic nuclei in the brains of all tested prion diseases, accompanying with severe spongiform degenerations in sCJD and G114V gCJD (middle panel). GFAP-specific IHC assays identified remarkable astrogliosis in the brains of sCJD, G114V gCJD, FFI Case 1 and 2, but less in FFI Case 3 (lower panel). Taking together with the presences of PrP^Sc^ (Figure 1), it seems that the activation of brain microglia does not correlate with the severity of any prion associated neuropathology, such as spongiform degeneration, neuron loss, astrogliosis and PrP^Sc^ deposit, but specially correlates with the subtypes of human prion diseases, i.e., sCJD.

### More inflammatory cytokines released in the brains of sCJD cases

**Figure 6:**
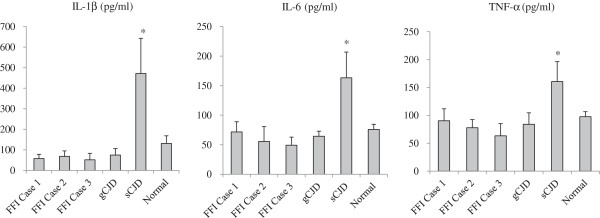
**Analyses of the levels of cytokines in temporal lobes of three FFI cases, a sCJD (Case 1) patient, a G114V gCJD patient and normal control.** Cytokines IL-1β, IL-6 and TNF-α in the brain homogenates were individually measured by the respective ELISA kits. The results are calculated from three independent tests and presented as mean ± SD. *: P<0.05.

To address the functional situation of brain microglia in various human prion diseases, the levels of several cytokines including IL-1β, IL-6, and TNF-α in the homogenates prepared from temporal lobes were quantitatively measured by ELISA kits. In the same amount of brain extracts, the levels of IL-1β, IL-6 and TNF-α in the brain tissues of sCJD were significantly upregulated (P<0.05), while the levels of the tested cytokines in the brains of FFI and G114V gCJD maintained at the baseline as the normal control (Figure 6). It reflects a functionally activated situation of microglia during the pathogenesis of sCJD.

## Discussion

Using Western blots, IFA and IHC, we have confirmed an almost silent proliferation of microglia in the brain tissues of human genetic prion diseases, including FFI and G114V gCJD, but an active proliferation of microglia in the brains of sCJD. Morphologically, the brain microglia cells change their cell sizes from small cell bodies with a few fibrils in normal control and genetic prion diseases to larger amoeboid soma with numerous fibers in sCJD cases. Moreover, the levels of several cytokines, including IL-1β, IL-6 and TNF-α, in the brains of sCJD show significantly increased, possibly reflecting an activated situation of microglia, while those in the brains of FFI cases and G114V gCJD case maintain comparable as normal control. Our data here may highlight distinct responses of CNS tissues when exposing to various subtypes of prions.

Human genetic prion diseases consist of three subtypes, gCJD, FFI and GSS. Although all of them are dominant genetic diseases, they may vary largely in the clinical manifestations, neuropathological features and PrP^Sc^ characteristics, due to the different mutations in *PRNP* and the polymorphism of the amino acid at codon 129. Previous literatures have repeatedly identified upregulation in microglial response in GSS, where microglial activation often accompanies prion protein deposition and neuronal loss [3,7,8]. Unlike GSS characterized pathologically by large amounts of amyloid deposits that mostly localize in the cerebral and cerebellar cortices and the basal ganglia, FFI usually lacks of observable PrP^Sc^ deposit, instead of extensive gliosis in thalamus, which might associate with the inactivation of microglial cells in FFI brains. Interestingly, although obviously PrP^Sc^ deposits are detected in the brains of G114V gCJD in our study, the brain microglial cells seems to be silent. The exact reason remains unknown. It cannot be excluded that the presence of different subtypes of prion strains among the various genetic prion diseases might affect the activation of microglia, irrespective of the presence of amyloid deposits Actually, in a set of animal models of CJD, different microglial activations have been already observed [9].

As resident immune cells in CNS, microglia may serve as an agent of immune surveillance and host defense that sensitively responses to the microenvironmental changes induced by neuronal injuries and infections [10]. Microglia activations in CNS tissues have been repeatedly identified in naturally-occurred scrapie in sheep and many scrapie-infected rodent models [11-15], as well as in bovine spongiform encephalopathy [16,17]. Similarly, activated microglial cells are also observed in the brain tissues, especially in the plaques of human infectious prion diseases, such vCJD and Kuru [18,19]. Meanwhile, microglia/macrophage induced inflammatory cytokines are increasingly released in the CNS tissues of various infectious human and animal TSEs [15,20]. Those data adequately illustrate that the host local immune defense system is activated during prion infections, by prion itself or/and amyloid plaques. Activations of microglia in CNS are also frequently observed in sCJD patients [6,19,21-23], though one study has proposed that less microglial cells are detected in the plaques of sCJD than those in vCJD and GSS, even less than Kuru [19]. The exact etiology of sCJD remains still unsettled, although it is usually considered due to the spontaneously conversion from PrP^C^ to PrP^Sc^ with unknown reason. However, the contact of exogenous infectious agent during long life-span of human being for sCJD cannot be absolutely excluded so far. Contrast to the acquired and sporadic forms of human prion diseases, the inherited human prion diseases, such as FFI and G114V gCJD in this study, are caused by the special mutated PrPs that usually show little infectivity in bioassays. One may speculate that besides of the formation amyloid plaque (for GSS and Alzheimer’s disease, AD), the subtypes of infectious human prions may also contribute to activation of microglia in CNS. In line with the description previously [6], our data here also show more activated microglia in sCJD with type 1 PrP^Sc^.

Besides of deposits of PrP^Sc^, neuron loss, astrogliosis and spongiform degeneration are also hallmarks for TSEs. However, those markers seem not to be associated with the level and status of microglial cells in the brains of FFI and G114V gCJD cases. More PrP^Sc^ deposits and more extensive spongiform have been observed in the cortex regions of the G114V gCJD case [24], while more severe gliosis have been observed in the regions of thalamus in those three FFI cases [25]. However, the levels of Iba1 positive signals among the ten tested brain regions from either FFI or G114V gCJD are quite comparable. No detectable plaque and extremely low amounts of PrP^Sc^ (PK-resistant PrP in Western blots) in FFI cases may relate to silent brain microglia. The brain tissues of G114V gCJD contain large amounts of PrP^Sc^ that is almost comparable with that of sCJD, but appear very limitedly increased microglia. It indicates again that the activation of microglia during prion pathogenesis may vary depending on the prion strains. Moreover, in addition to its effect of agent clearance, activation microglia also possibly contributes to enhance the neuronal destruction [9]. Apoptotic neurons in CJD are probably related to the presence of inflammatory cells and cytokines which are present during the whole CJD disease process [26]. Lack of or very limited activated microglia in the CNS tissues of FFI and G114 gCJD suggest that recruitment of inflammatory cells is not the major reason for neuronal destruction for these two inherited prion diseases, which highlight again the diversity of the pathogenesis of human prion diseases [1,27].

## Conclusion

The paper concludes that the brain microglia is relatively silent in FFI and G114V gCJD, but obviously activated in sCJD, which reflects various pathogenesis of different human prion diseases subtypes.

## Methods

### Brain samples from human prion diseases and normal control

The stored frozen brain tissues and paraffin-embed brain tissues from three FFI cases, two sCJD cases and one G114V gCJD case were enrolled in this study. All cases were definitely diagnosed human CJD cases based on the diagnostic criteria for Creutzfeldt-Jakob disease issued by WHO. The clinical features and the neuropathological abnormalities of three FFI cases and the G114V gCJD case were separately described previously [24,25]. The presences of PrP^Sc^ in the brain tissues of two sCJD patients were confirmed by the proteinase K (PK) treated Western blots with PrP specific antibody. The death ages of those patients were 48 (FFI Case 1), 26 (FFI Case 2), 55 (FFI Case 3), 47 (G114V gCJD), 63 (sCJD Case 1) and 81 (sCJD Case 2) years old, respectively. Normal human brain tissues were obtained from a health donor died from car accident at the age of 56.

### Preparation of brain homogenates and western blots

Brain tissues from normal and prion diseases were washed with iced TBS (10 mM Tris HCl, 133 mM NaCl, pH 7.4). 10% (w/v) brain homogenates were prepared based on the protocol described previously [28]. Briefly, brain tissues were homogenized in lysis buffer (100 mM NaCl, 10 mM EDTA, 0.5% Nonidet P-40, 0.5% sodium deoxycholate, 10 mM Tris, pH 7.5) containing a mixture of protease inhibitors. Tissue debris was removed with low speed centrifugation at 2000 g for 10 min and the supernatants were collected for further study. Aliquots of brain homogenates were separated on 12% SDS-PAGE and electroblotted onto a nitrocellulose membrane using a semi-dry blotting system (Bio-Rad). Membranes were blocked with 5% (w/v) non-fat milk powder (NFMP) in 1 × Tris-buffered saline containing 0.1% Tween 20 (TBST) at room temperature (RT) for 1 h and probed with individual primary antibodies at 4°C overnight, including rabbit anti-Iba1 pAb (Wako, 019–19741), anti-PrP mAb (3F4, Chemicon, MAB1562) and anti-β-actin mAb (Subrray Biotechnology, Sr-25113), respectively. After washing with TBST, blots were incubated with horseradish peroxidase (HRP)-conjugated goat anti-mouse or rabbit IgG (Jackson ImmunoResearch Labs, 115-035-003, 111-035-003), at RT for 2 h. Blots were developed using Enhanced ChemoLuminescence system (ECL, PerkinElmer, NEL103E001EA) and visualized on autoradiography films. Images were captured by ChemiDoc™ XRS^+^ Imager (Bio-Rad).

To detect the presences of PK resistant PrP^Sc^ in brain tissues, the brain homogenates were firstly digested with a final concentration of 20 μg/ml PK at 37°C for 60 min prior to Western blots. The PK digestion was terminated by heating the samples at 100°C for 10 minutes.

### Immunohistochemical (IHC) assays

Brain tissue was fixed in 10% buffered formalin solution. Before histological processes, all the fixed tissues were immersed in 98% formic acid for at least 1 h for inactivation of infection. Paraffin sections (5 μm in thickness) of brain tissues were prepared and IHC assays were performed according to published protocol [28]. Prior to the staining of PrP mAb, brain sections were treated by 6M GdnHCl for 10 min. Sections were quenched for endogenous peroxidases in 3% H_2_O_2_ in methanol for 10 min, pretreated with enzyme digestion antigen retrieval for 1 min. After blocking in 1% normal goat serum, the sections were incubated overnight at 4°C with rabbit anti-Iba1 pAb, anti-PrP mAb (3F4) or 1 anti-GFAP mAb, respectively. The sections were then incubated with HRP-conjugated goat anti-mouse or rabbit secondary antibody (Boster, SV0001-12 or SV0002-12) for 60 min, and visualized by incubation with 3,3-diaminobenzidine tetrahydrochloride (DAB). The slices were counterstained with hematoxylin, dehydrated and mounted in permount.

### Nissl staining and HE staining

Brain paraffin sections (5 μm in thickness) of temporal lobes from various human prion diseases were analyzed by routine HE staining and Nissl staining. For Nissl staining, slices were stained with Nissl (1% toluidine blue) for 30 min based on the protocol described elsewhere. The slices were mounted with permount and observed under a light microscopy.

### Immunofluorescence and confocal microscopy assays

Prior to the staining of PrP mAb, brain slides were treated with 6M GdnHCl for 10 minutes. The sections were blocked in 1% normal goat serum and then incubated with a mixture of rabbit anti-Iba1 pAb and anti-PrP mAb 3F4 at 4°C overnight. Subsequently, the sections were incubated with Alexa Fluor 586-labeled goat anti-rabbit and Alexa Fluor 488-labeled goat anti-mouse secondary antibody (Invitrogen, A-11037 or A11029) for 60 min. Finally, the sections were incubated with DAPI (Invitrogen, D1306) for 10 min. The slices were mounted in permount and analyzed by confocal microscopy (Leica ST2, Germany).

### Preparation of brain lysates and ELISAs

The brain samples stored at −80°C were homogenized in lysis buffer (150mM NaCl, 1% TritonX-100, 10mM Tris, PH 7.4) containing proteinase inhibitor. The homogenates were centrifuged at 10,000 g for 10 min at 4°C. Supernatants were collected and aliquoted after measuring their protein concentration using a bicinchoninic acid (BCA) protein assay kit (Novagen, 71285–3), and adjustment to 500 μg/μl. The lysates were stored at −80°C for further analysis.

ELISA kits were used to detect the following cytokines: IL-1β, IL-6 and TNF-α (Boster, EK0390; Boster, EK0393; Boster, EK0412; TBD science, KIT0003). Assays for cytokines in brain lysates were performed according to the manufacturer’s protocol. Briefly, individual lysates were 10-fold diluted with the blocking buffer. 100 μl of diluted preparations, as well as standard samples, were added to each well and incubated at RT for 2 h. After washing with 300 μl of provided wash buffer for 4 times, 100 μl biotin-conjugated antibodies were added and incubated at RT for 1 h, and subsequently incubated with HRP-conjugated streptavidin at RT for 30 min. Finally, substrate solution (0.05% 3,3’,5,5’-tetramethybenzidine and 0.012% H_2_O_2_ in 0.05 M citrate buffer, pH 5.0) was added and incubated at 37°C for 20 min and stopped with 1M H_2_SO_4_. The optical density at λ=450 nm was measured in a microplate reader (Thermo), and the cytokine concentrations of brain lysates were calculated with the help of the calibration curve generated by using known amounts of standards.

### Statistical analysis

Statistical analysis were performed using *SPSS* 17.0 statistical package. Quantitative analysis of immunoblot images were carried out using software Image J. The gray values of each target blot were evaluated. All data were presented as the mean ± SD. Statistical analyses were performed using Student’s *t* test. *P* value less than 0.05 was considered to be statistically significant.

### Ethical statement

This study was approved by the Ethical Committee of National Institute for Viral Disease Control and Prevention, China CDC under protocol 2009ZX10004-101.

## Competing interests

The authors declare that they have no competing interests.

## Authors’ contributions

QS and WLX carried out all experiments in this study, collated the information, performed the literature search and drafted the manuscript. BYZ performed the animal experiment. LNC, KW and CC assisted to perform the neuropathological assays and animal tests. YX performed the ELISA experiment. KR and XMZ assisted to finish the Western blots. JZ performed the immunofluorescence and confocal microscopy assays. XPD, the corresponding author, designed the research project, performed the literature search and prepared the manuscript. All authors read and approved the final manuscript.
